# Choosing Wisely Canada – pediatric otolaryngology recommendations

**DOI:** 10.1186/s40463-021-00533-x

**Published:** 2021-10-29

**Authors:** Mitchell McDonough, Kalpesh Hathi, Gerard Corsten, Christopher J. Chin, Paolo Campisi, Jonathan Cavanagh, Neil Chadha, M. Elise Graham, Murad Husein, Liane B. Johnson, Jodi Jones, Bruce Korman, John Manoukian, Lily H. P. Nguyen, Doron D. Sommer, Julie Strychowsky, Trina Uwiera, Warren Yunker, Paul Hong

**Affiliations:** 1 Dalhousie Medicine New Brunswick, Saint John, New Brunswick Canada; 2 Division of Otolaryngology - Head & Neck Surgery, Department of Surgery, Dalhousie University, 5850/5920 University Ave, Halifax, Nova Scotia B3K 6R8 PO Box 9700 Canada; 3 Department of Otolaryngology - Head & Neck Surgery, University of Toronto, Toronto, Ontario Canada; 4 Division of Otolaryngology - Head & Neck Surgery, Department of Surgery, Memorial University of Newfoundland, St. John’s, Newfoundland and Labrador, Newfoundland, Canada; 5 Division of Otolaryngology - Head & Neck Surgery, Department of Surgery, University of British Columbia, Vancouver, British Columbia Canada; 6 Department of Otolaryngology - Head & Neck Surgery, Western University, London, Ontario Canada; 7 Department of Otolaryngology - Head & Neck Surgery, University of Manitoba, Winnipeg, Manitoba Canada; 8 Department of Surgery, Otolaryngology/Head & Neck Surgery Division, McMaster University, Hamilton, Ontario Canada; 9 Division of Otolaryngology - Head & Neck Surgery, Department of Surgery, McGill University, Montreal, Quebec, Canada; 10 Division of Otolaryngology - Head & Neck Surgery, Department of Surgery, University of Alberta, Edmonton, Alberta Canada; 11 Section of Otolaryngology - Head & Neck Surgery, Department of Surgery, University of Calgary, Calgary, Alberta Canada

**Keywords:** Pediatric otolaryngology, Choosing Wisely Canada, Health care utilization, Best practice, Otitis media with effusion, Tonsillectomy

## Abstract

The Choosing Wisely Canada campaign raises awareness amongst physicians and patients regarding unnecessary or inappropriate tests and treatments. Using an online survey, members of the Pediatric Otolaryngology Subspecialty Group within the Canadian Society of Otolaryngology – Head & Neck Surgery developed a list of nine evidence based recommendations to help physicians and patients make treatment decisions regarding common pediatric otolaryngology presentations: (1) Don’t routinely order a plain film x-ray in the evaluation of nasal fractures; (2) Don’t order imaging to distinguish acute bacterial sinusitis from an upper respiratory infection; (3) Don’t place tympanostomy tubes in most children for a single episode of otitis media with effusion of less than 3 months duration; (4) Don’t routinely prescribe intranasal/systemic steroids, antihistamines or decongestants for children with uncomplicated otitis media with effusion; (5) Don’t prescribe oral antibiotics for children with uncomplicated tympanostomy tube otorrhea or uncomplicated acute otitis externa; (6) Don’t prescribe codeine for post-tonsillectomy/adenoidectomy pain relief in children; (7) Don’t administer perioperative antibiotics for elective tonsillectomy in children; (8) Don’t perform tonsillectomy for children with uncomplicated recurrent throat infections if there have been fewer than 7 episodes in the past year, 5 episodes in each of the past 2 years, or 3 episodes in each of the last 3 years; and (9) Don’t perform endoscopic sinus surgery for uncomplicated pediatric chronic rhinosinusitis prior to failure of maximal medical therapy and adenoidectomy.

## Introduction

Delivery of efficient healthcare has become increasingly important. The Choosing Wisely Canada campaign raises awareness amongst physicians and patients regarding unnecessary or inappropriate tests and treatments. The Canadian Society of Otolaryngology – Head & Neck Surgery (CSOHNS) is a proud partner of Choosing Wisely Canada and has previously released recommendations for Head and Neck Surgical Oncology [1], Otology/Neurotology [2], and Rhinology [3].

The CSOHNS is dedicated to improving patient care through the support of education, promotion of research, dissemination of information, scientific advancement of the society, and the maintenance of high professional and ethical standards. The Pediatric Otolaryngology Subspecialty Group (POSG) of the CSOHNS maintains these same core values and strives to promote evidence-based care amongst physicians and patients through the development of a Choosing Wisely Canada recommendation list.

Pediatric otolaryngology complaints are commonly seen in many different settings. These recommendations aim to reduce unnecessary or inappropriate tests or treatments and may be relevant to various providers including general practitioners and otolaryngologists encountering pediatric otolaryngology patients.

## Methods

The current list was created by members of the POSG of the CSOHNS. Members were chosen based on their ongoing affiliation with an academic teaching hospital and their subspecialty training in pediatric otolaryngology. An initial list of 25 recommendations regarding unnecessary tests and interventions were compiled, along with sourced material to support the recommendations. Members of the group, considered to be national leaders within the subspecialty, were then asked to complete an electronic survey providing feedback on each recommendation and ranking their merit. The survey was constructed using Opinio (ObjectPlanet Inc., Oslo, Norway).

Each recommendation was rated by individual PSOG members based on five commonly evaluated factors: potential to affect clinical practice, cost-benefit ratio, potential to cause harm, evidence supporting recommendation, and pervasiveness of test/intervention [4]. A scale of 1–9 was used, with 1–3 indicating low impact/not relevant, 4–6 as uncertain, and 7–9 indicating high impact/high relevance. In addition, each recommendation had a comment section for respondents to input feedback, as well as a question asking if the recommendation should be included in the final list (yes, no, uncertain). The results of the survey were used to rank the 25 recommendations based on highest overall score, and comments were used to revise or reject certain recommendations. Based on this, nine recommendations had general consensus of merit and moved on to a panel review for final editing. The list was circulated to members of the POSG for approval. These recommendations were peer reviewed and subsequently released on the Choosing Wisely Canada website, as well promoted through Twitter and an email campaign.

## Results

### Don’t routinely order a plain film x-ray in the evaluation of pediatric nasal fractures

Nasal fractures are one of the most common facial fractures in the pediatric population [5]. The decision to perform a closed reduction procedure for an uncomplicated nasal fracture in the operating room is based on factors such as breathing difficulty and external deformity, which are not assessed effectively by x-ray [6]. Plain film x-rays are unable to accurately evaluate nasal fractures given its low sensitivity and specificity, at 72 and 73% respectively [6]. Physical examination is often sufficient to make a diagnosis for children with displaced nasal fractures [7]. Overall, x-rays do not add value to the diagnosis or treatment plan for children with nasal fractures and should not be ordered to avoid their associated costs and radiation exposure.

### Don’t order imaging to distinguish acute bacterial sinusitis from an upper respiratory infection

Uncomplicated acute bacterial sinusitis (ABS) is a diagnosis that is made based on clinical criteria and has a low prevalence amongst children presenting with respiratory symptoms [8–10]. Although a normal x-ray, CT, or MRI can help to rule out ABS, an abnormal result does not confirm the diagnosis [8]. Given that many children will have abnormal findings on imaging due to viral upper respiratory infections and/or other inflammatory diseases during certain times of the year, combined with the potential for radiation exposure, imaging is not routinely recommended [11, 12]. Instances in which imaging would be warranted include if the child is immunocompromised, or if orbital, central nervous system, or suppurative complications are present [8].

The American Academy of Pediatrics recommends diagnosing pediatric ABS when a child with an acute upper respiratory tract infection presents with (1) persistent illness (nasal discharge [of any quality] or daytime cough or both lasting more than 10 days without improvement), (2) a worsening course (worsening or new onset of nasal discharge, daytime cough, or fever after initial improvement), or (3) severe onset (concurrent fever [temperature ≥ 39 °C/102.2 °F] and purulent nasal discharge for at least 3 consecutive days) [8].

### Don’t place tympanostomy tubes in children for a single episode of uncomplicated otitis media with effusion of less than 3 months’ duration

Although tympanostomy tube insertion can be associated with short-term quality of life improvements, the natural history of otitis media with effusion (OME) is sufficiently favorable and the majority of uncomplicated OME cases in children will spontaneously resolve within 3 months [13–15]. Cases of OME lasting longer than 3 months are typically chronic in nature, and less likely to resolve without intervention [13, 16]. Limited data exists regarding the efficacy of tympanostomy tube insertion in children with OME for less than 3 months. By delaying the consideration for tympanostomy tube insertion, potentially unnecessary procedures are avoided, along with the associated risks, tube related side effects, and costs [16]. Children who present with a single episode of uncomplicated OME should be reassessed at 3 months and 6 months for indications for intervention [13]. Children excluded from this recommendation include those who have risk factors for developmental difficulties such as trisomy 21, autism-spectrum disorder, craniofacial disorders, blindness, other developmental delays and permanent hearing loss independent of OME [13].

### Don’t routinely prescribe intranasal/systemic steroids, antihistamines or decongestants for children with uncomplicated otitis media with effusion

In most cases, medical treatment using antihistamines, decongestants, systemic antibiotics and steroids have shown little to no effect on the long-term outcomes of uncomplicated OME in children [17–19]. In addition, there are associated costs and potential side effects of these medications [20]. Therefore, the recommendation is not to routinely prescribe these medical treatments for children with uncomplicated OME. The exception to this would be for children with coexisting conditions in which these medications are indicated for primary management (e.g., children with allergic rhinitis) [17].

### Don’t prescribe oral antibiotics for children with uncomplicated tympanostomy tube otorrhea or uncomplicated acute otitis externa

The use of unnecessary oral antibiotics can promote antibiotic resistance and increase the risk of opportunistic infections [21]. Topical antibiotics achieve higher concentrations in the ear canal and middle ear, demonstrate improved patient satisfaction, are associated with fewer adverse events, and are shown to have equal efficacy for treatment of acute tympanostomy tube otorrhea (TTO) and acute otitis externa (AOE) when compared to oral antibiotics [21–25]. For these reasons, topical antibiotics rather than oral antibiotics should be prescribed as first line treatment for acute uncomplicated TTO and uncomplicated AOE.

### Don’t prescribe codeine for post-tonsillectomy/adenoidectomy pain relief in children

Codeine has been associated with a high rate of adverse drug reactions in children [26, 27]. This includes life-threatening respiratory depression [28, 29]. Appropriate dosing of codeine is challenging due to the genetic heterogeneity amongst patients for the CYP2D6 enzyme, which is responsible for codeine metabolism [26, 30]. Genetic screening of CYP2D6 is not routinely performed and cannot reliably identify variations in codeine metabolism rates among patients [26, 30]. As such, children who are ultra-fast metabolizers of codeine are placed at increased risk of severe adverse drug reactions [28, 29]. Alternative analgesia should be used post-tonsillectomy/adenoidectomy. Providers should be aware that other analgesics could also have active metabolites which may be of concern in children at an inappropriate dose (e.g., morphine).

### Don’t administer perioperative antibiotics for elective tonsillectomy in children

Administration of perioperative antibiotics for children undergoing elective uncomplicated tonsillectomy shows no significant benefits in regard to common post-tonsillectomy morbidities [31, 32]. Overuse of systemic antibiotics increases bacterial resistance and the risk of adverse drug events unnecessarily. These concerns outweigh the reduction in postoperative fever which is the only potential benefit of perioperative antibiotic administration for elective tonsillectomy [31, 32]. Therefore, perioperative antibiotics are not recommended for children undergoing elective tonsillectomy, unless specific indications are present (e.g., cardiac conditions or those with a peritonsillar abscess or active infection) [31].

### Don’t perform tonsillectomy for children with uncomplicated recurrent throat infections if there have been fewer than 7 episodes in the past year, 5 episodes in each of the past 2 years, or 3 episodes in each of the last 3 years

For children who have a lower number of recurrent throat infections, tonsillectomy has significantly less benefit when compared to those with more frequent infections, and many children with recurrent throat infections naturally improve without intervention [33, 34]. Therefore, where safely possible, avoidance of tonsillectomy for children with lower number of acute infections is recommended [35]. This avoids unnecessary tonsillectomy and the costs and complications associated with the procedure (e.g., bleeding, pain, infection) [33, 34]. If tonsillectomy is not indicated, children should be closely monitored and reconsidered for tonsillectomy if the infection frequency increases, as they would be less likely to naturally improve, and more likely to benefit from tonsillectomy. Families should be counselled on the limited benefits and potential harms of performing tonsillectomy for children and adolescents with low rates of recurrent throat infections [35]. Shared decision making is of importance when considering tonsillectomy as individual patient and family factors can impact the decision. Providers should take into account the clinical features and severity of throat infections and social factors (e.g., missed school days) relevant to the individual patient and family. Exceptions to this recommendation include patients with recurrent peritonsillar abscesses, PFAPA (Periodic Fever, Aphthous Stomatitis, Pharyngitis, Adenitis), severe infections, rheumatic heart disease or Lemierre’s syndrome [35].

### Don’t perform endoscopic sinus surgery for uncomplicated pediatric chronic rhinosinusitis prior to failure of maximal medical therapy and adenoidectomy

While endoscopic sinus surgery (ESS) has been found to be an effective therapy in children with uncomplicated chronic rhinosinusitis, comparable outcomes can be achieved with medical therapy and adenoidectomy [36, 37]. A stepwise approach of medical therapy, progressing to adenoidectomy, then to ESS allows children to be treated with less invasive and more cost-effective interventions as initial therapy, while saving ESS for those who are refractory to primary interventions [37, 38]. Maximal medical therapy should be exhausted prior to surgical intervention for uncomplicated patients [37–39]. In cases with complications such as orbital or skull base involvement, ESS can be employed more readily.

## Discussion

These recommendations are not hard “rules” but are rather intended to promote discussion amongst physicians and patients regarding evidence-based approaches to reduce inappropriate or unnecessary treatment for pediatric otolaryngology patients. Relevance for patients revolves primarily around providing a concise description as to why practitioners may opt for or against certain treatments. In addition to these recommendations being open access on their website, Choosing Wisely Canada actively promotes their campaigns through social media and posters for use in clinicians’ offices, both of which patients may be exposed to. Clinicians should carefully tailor treatments and discussions with patients based on each individual patient’s unique presentation and circumstances. These recommendations are not meant to establish payment or coverage by insurers.

## Data Availability

Not applicable.

## Abbreviations

ABS: Acute Bacterial Sinusitis
AOE: Acute Otitis Externa
CT: Computed Tomography
CSOHNS: Canadian Society of Otolaryngology- Head & Neck Surgery
ESS: Endoscopic Sinus Surgery
MRI: Magnetic Resonance Imaging
OME: Otitis Media with Effusion
PFAPA: Periodic Fever, Aphthous Stomatitis, Pharyngitis, Adenitis
POSG: Pediatric Otolaryngology Subspecialty Group
TTO: Tympanostomy Tube Otorrhea
